# Neuroprotective Effects of the Herbal Formula B401 in Both Cell and Mouse Models of Alzheimer's Disease

**DOI:** 10.1155/2016/1939052

**Published:** 2016-09-28

**Authors:** Chih-Hsiang Hsu, Sheue-Er Wang, Ching-Lung Lin, Chun-Jen Hsiao, Shuenn-Jyi Sheu, Chung-Hsin Wu

**Affiliations:** ^1^Department of Life Sciences, National Taiwan Normal University, Taipei City, Taiwan; ^2^Department of Pathological Inspection, Saint Paul's Hospital, Taoyuan City, Taiwan; ^3^Brion Research Institute of Taiwan, New Taipei City, Taiwan

## Abstract

In this study, we have reported the herbal formula B401 that has neuroprotective effects via multifunction, multitarget characteristics. It is possible that the herbal formula B401 may also provide new insights for AD. Here, we studied protective effects in the Tet-On A*β*_42_-GFP SH-SY5Y cell model and the APP/PS1/Tau triple transgenic mouse model by the herbal formula B401. In* in vitro* experiments, we showed that the herbal formula B401 treatment effectively reduces glutamate-induced excitotoxicity and acetylcholinesterase activity in Tet-On A*β*_42_-GFP SH-SY5Y cells. In* in vivo* experiments, we found that oral B401 treatment effectively ameliorates neurocognitive dysfunctions of 3× Tg-AD mice via motor and cognitive behavior tests. By using magnetic resonance imaging, moorFLPI instruments, and chemiluminescence methods, we reported that oral B401 treatment effectively alleviates brain atrophy, improves subcutaneous blood flow, and reduces blood ROS in 3× Tg-AD mice. As observed from results of immunohistochemistry staining and western blotting, we found that oral B401 treatment significantly enhances expressions of neuroprotective proteins, while reducing expressions of AD derived proteins such as amyloid beta, phosphorylated Tau, neurofibrillary tangles, and 3-nitrotyrosine in the brain of 3× Tg-AD mice. Thus, the herbal formula B401 may have the potential to be developed into optimum TCM for AD patients.

## 1. Introduction

Alzheimer's disease (AD) is a chronic neurodegenerative disease with obvious memory loss. AD hallmarks such as amyloid plaques and neurofibrillary tangles (NFTs) are obviously found in the brains of AD patients [1, 2]. The amyloid plaques are abnormal clusters of dead nerve cells and beta amyloid (A*β*) proteins, while the NFTs are twisted protein fragments inside the nerve cells. These amyloid plaques and NFTs prevent neurons from communicating with other neurons and hence cause the cognitive deficits in the brain. Intracellular A*β* aggregation leads to the hyperphosphorylation of Tau, the disruption of mitochondria function, and the synapse dysfunction [3–6]. In addition, NFTs are abnormal heaps of phosphorylated Tau proteins [7]. Tau protein is a soluble microtubule-binding protein that can attach and stabilize microtubules contributing to axonal transport and neurite outgrowth [8, 9]. In addition, Tau is hyperphosphorylated leading to its detachment from microtubules and subsequently the formation of soluble Tau aggregates and NFTs [10].

AD patients are clinically diagnosed with a progression from episodic memory and learning ability deficits to the decline of cognitive function and have average 9 years of life span after diagnosis [11, 12]. The conventional therapy for mild to moderate AD symptoms is the treatment with AChE inhibitors such as memantine to have better cognitive function [13, 14]. It is possible that decreasing acetylcholine level in AD brain may lead to cognitive impairment [15–17]. Acetylcholinesterase (AChE) is an enzyme that catalyzes acetylcholine hydrolysis and is mainly found at cholinergic brain synapses and neuromuscular junctions to terminate synaptic transmission. Nowadays, the medication for AD is on the basis of AChE inhibitors to improve cholinergic functions in AD patients [17–19]. However, until now, markedly clinical therapies for neurodegeneration of AD remain elusive.

Alternative medical applications of traditional Chinese medicines (TCMs) in treating neurodegenerative disease are becoming popular because of their clinical safety. Particularly TCMs in the form of formulas may produce synergistic effects and reduce side effects of drug toxicity. As suggested from many studies of alternative medicine, traditional Chinese medicines provide new insights for neurodegenerative disease. For example, the herbal formula B401 is a famous patent TCM that was widely used in Taiwan as a health supplement in supporting brain healthy and cardiovascular function. We have reported that the herbal formula B401 may provide a possible clinical therapy in R6/2 transgenic mice of Huntington's disease [20, 21]. We found that oral herbal formula B401 treatment can enhance brain-derived neurotrophic factor (BDNF) in the brain tissue of these R6/2 transgenic mice. Coincidentally, obviously reduced BDNF levels were also found in the brain of AD patients [22–25]. It is possible that the herbal formula B401 may alleviate neuropsychiatric symptoms in AD patients via enhancing BDNF levels in their brain.

To study potential therapeutic agent for AD, several cell and animal models have been generated to develop AD-like pathology. In the present study, we aimed at the neuroprotective potential of the herbal formula B401 in AD. To approach our aims, we selected a Tet-On A*β*_42_-GFP SH-SY5Y cell model and a APP/PS1/Tau triple transgenic AD (3× Tg-AD) mouse model to assess the beneficial use of the herbal formula B401 in complimentary or integrated therapy for neuroprotective and neuropsychiatric remission in AD. The 3× Tg-AD mice are generated to mimic the pathology of AD and successfully developed both amyloid plaque and NFTs-like pathology [26–31]. Behavioral characterization of 3× Tg-AD mice reveals a reduction of exploratory activity, as well as learning and memory deficits. Moreover, 3× Tg-AD mice may exhibit higher sensitivity and anxiety than normal mice. With aids of Tet-On A*β*_42_-GFP SH-SY5Y cell model and a 3× Tg-AD mouse model, possible neuroprotective potential of the herbal formula B401 for AD was clarified in this study.

## 2. Materials and Methods

### 2.1. Preparation of the Herbal Formula B401

The chromatographic fingerprint analysis of the herbal formula B401 (provided by Brion Research Institute of Taiwan) was conducted by using LC/MS (liquid chromatography/mass spectrometry) analysis. The LC/MS analytical system used in this study was the combination of a LC-20AD UFLC system (Shimadzu Corporation, Kanagawa, Japan) linked with a LCMS-8040 triple quadrupole mass spectrometer (Shimadzu Corporation).

### 2.2. MTT Assay

Human neuroblastoma Tet-On A*β*_42_-GFP SH-SY5Y cells viability was measured by MTT (3-(4,5-dimethylthiazol-2-yl)-2,5-diphenyltetrazolium bromide) assay. Tet-On A*β*_42_-GFP SH-SY5Y cells were generously supplied by Dr. Guey-Jen Lee-Chen at National Taiwan Normal University (NTNU). Previously A*β*_42_ was fused to the N-terminus of GFP to couple the aggregation state with the fluorescence of GFP. Inhibitors that retard or block A*β* aggregation can be distinguished by increasing fluorescence on Tet-On 293 cells; thus A*β*_42_-GFP could be used to generate Tet-On 293 cell clone as screening platforms. In this study, Tet-On A*β*_42_-GFP SH-SY5Y neuroblastoma cells were plated in the 6-well plate at a density of 3.0 × 10^4^ cells/well with 10 *μ*M retinoic acid (Sigma-Aldrich Corporation) and pretreated with 10, 20, 40, 80, and 160 mg/mL for 24 hours and then induced with 10 *μ*g/mL doxycycline (Dox, Sigma-Aldrich Corporation) to express A*β*_42_-GFP for five days. All cell viability assay was approved by the NTNU Committee on Biological Research.

### 2.3. AChE Activity Assay

Tet-On A*β*_42_-GFP SH-SY5Y neuroblastoma cells in the absence or presence of the herbal formula B401 were harvested with cold phosphate buffered saline (PBS, Falcon Inc., McLean, VA, USA), followed by sonication and centrifugation at 13,000 rpm for 20 min at 4°C, and the supernatants were collected for AChE activity assay (AChE assay kit purchased from Thermo Fisher Scientific Inc., Waltham, USA). 10 *μ*L samples were transferred into separate wells of a 96-well plate, and 190 *μ*L of fresh Working Reagent was added to all sample wells. 200 *μ*L water and 200 *μ*L calibrator were transferred to individual wells. The absorbance was measured at OD 412 nm with an ELISA reader at 2 min and 10 min.

### 2.4. Animals Preparation

The triple transgenic mouse model of AD (3× Tg-AD transgenic mouse) harboring human PS1M146V, human APPswe, and human tauP301L was purchased from the Jackson Laboratory (Sacramento, California, USA). C57BL/6 mice as a control group were purchased from National Laboratory Animal Center (NLAC, Taipei, Taiwan). All mice were housed in a temperature-controlled environment at 22°C ± 2°C with a 12-hour light/dark cycle and food* ad libitum*. 3× Tg-AD transgenic mice were further divided into two groups: the first group was 8 3× Tg-AD mice with oral DMSO treatment (AD mice with sham treatment) and the second group was 8 3× Tg-AD mice with oral B401 treatment at a daily dose of 50 mg/kg (AD mice with B401 treatment), once every other day from 6 months to 8 months of age. Then 8-month C57BL/6 (non-AD) mice and 3× Tg-AD mice with sham and oral B401 treatment were applied for behavioral tasks. After completion of behavioral task, all mice were sacrificed and the brain tissues were dissected for western blotting as well as immunohistochemistry analyses. All animal experiments were approved by the Institutional Animal Care and Use Committee at NTNU (Protocol number: NTNU/Animal Use/No. 101020).

### 2.5. Morris Water Maze Test

As reported in the previous study [32], the Morris water maze is test in a circular pool (100 cm in diameter and 35 cm in height). The pool was equally divided into four quadrants and a white platform was submerged 1 cm below the surface of the water and centered in one of the four quadrants of the pool. The swimming path length to the platform, escape latency, and velocity were recorded by a computer-controlled system.

### 2.6. Spontaneous Alternation Behavior Y-Maze Test

As reported in the previous study [31], the Y-maze is a three-arm maze (30 cm long and 5 cm wide with 12 cm in height) with equal angles and the arms were labeled A, B, and C. We count the number of arm entries per trial as an indicator of locomotors. The percentage of alternation was calculated by the following equation: Alternation (%) = [(Number of alternations)/(Total arm entries − 2)] × 100.

### 2.7. Novel Object Recognition Task

As reported in the previous study [33], the apparatus was an open field (50 × 40 cm, with 22 cm in height) with white walls and floor and placed in a quiet room. The general procedure included three different phases (habituation phase, sample phase, and test phase). During the test phase, the mouse was placed back in the arena and exposed to two objects. The time spent exploring the objects was defined as the distance from nose to object within 1-2 cm or/and touching it with the nose and forepaws. The discrimination index was calculated as percentage ratio. A discrimination index of higher than 50% represents good cognitive performance.

### 2.8. Brain Morphology Analysis

Brain morphology of mice was analyzed with 7 T horizontal bore magnetic resonance imaging (MRI) system (Bruker BioSpec 70/30 USR; Bruker BioSpin Corporation, Billerica, MA, USA). With aid of Vitrea Core software (Toshiba Medical Systems, Minnetonka, MN, USA), horizontal and lateral ventricle sizes of brain were calculated.

### 2.9. Skin Blood Flow Analysis

As reported in our previous study [20], regional dermal microvascular blood flows of mice were scanned by laser Doppler imager (Moor Instruments, Axminster, UK). Then blood flow was calculated in arbitrary perfusion units with the aid of the data acquisition software (MoorFLPI measurement software, Version 3.0; Moor Instruments).

### 2.10. ROS Analysis

As reported in our previous study [21], O_2_^•−^ and H_2_O_2_ activity of mice were detected by lucigenin- and luminol-amplified chemiluminescence (CL) methods with aids of chemiluminescence analyzer (CLA-ID3; Tohoku Electronic Industrial Co., Ltd., Sendai, Japan).

### 2.11. Western Blot Analysis

After completion of the behavioral task, mice were anesthetized with urethane (1.5 mg/kg) and transcardially perfused with physiological saline. The brain tissue of mice was removed and homogenized in a buffer solution that was quantified by a BCA protein assay kit (Thermo Fisher Scientific Inc., Waltham, Massachusetts, USA). Then proteins of brain tissue were separated in SDS polyacrylamide gels (Bionovas Pharmaceuticals Inc., Washington DC, USA) and then were transferred to polyvinylidene difluoride membranes (GE Healthcare Life Sciences, Barrington, Illinois, USA). The antibodies utilized in this study were the brain-derived neurotrophic factor (BDNF) (Santa Cruz Biotechnology Inc.), vascular endothelial growth factor (VEGF) (Cell Signaling Technology Inc.), superoxide dismutase 2 (SOD2) (Cell Signaling Technology Inc.), 3-nitrotyrosine (3-NT) (Cell Signaling Technology Inc.), *β*-actin (Thermo Fisher Scientific Inc.), amyloid beta (A*β*) (Cell Signaling Technology Inc., Danvers, USA), phosphorylated Tau (p-Tau) (Ser396, Cell Signaling Technology Inc.), and neurofibrillary tangles (NFTs) (Cell Signaling Technology Inc.). Horseradish peroxidase- (HRP-) conjugated secondary antibody (Santa Cruz Biotechnology Inc.) was used to detect these antibodies. Then the enhanced chemiluminescence (ECL) substrate (Millipore, Billerica, Massachusetts, USA) was used to observe immunoreactive bands of these proteins. Finally, we quantified the band intensities of these proteins with the Image J analysis software (version 1.48t, Wayne Rasband, USA).

### 2.12. Immunohistochemistry

Anesthetized mice were cardiac perfused with phosphate buffered saline (PBS) containing 4% formaldehyde (Sigma-Aldrich Corporation), and then removed brain tissues were fixed with 4% formaldehyde (EM grade) (Sigma-Aldrich Corporation) and embedded in paraffin. These brain specimens were cut into tissue sections at a thickness of 5 *μ*m and were mounted on slides for histological and IHC stains. Hematoxylin and eosin (H&E) staining with a kit-based approach (Sigma-Aldrich Corporation) was used to histologically assess brain morphology of these mice. Furthermore, we used the heat-induced epitope retrieval method to assess IHC stains of brain tissue sections. Brain tissue sections were separately stained with antibodies of SOD2 (Cell Signaling Technology Inc.), 3-NT (Cell Signaling Technology Inc.), A*β* (Cell Signaling Technology Inc.), p-Tau (Cell Signaling Technology Inc.), and NFTs (Cell Signaling Technology Inc.) at room temperature for one hour. Then brain tissue sections were immunologically detected by biotinylated secondary antibodies (NovolinkTM polymer detection system l, Leica Biosystems Newcastle Ltd., Newcastle, United Kingdom) and then by avidin-biotin-HRP complex (NovolinkTM polymer detection system l, Leica Biosystems Newcastle Ltd.) for 30 minutes. Finally, IHC stains of these brain tissue were perceived by DAB Chromogen (NovolinkTM polymer detection system l, Leica Biosystems Newcastle Ltd.).

### 2.13. Statistical Analysis

Results were obtained from at least 3 independent experiments, all data was given as mean ± SEM. The data was analyzed with one-way or two-way ANOVA followed by Student-Newman-Keuls multiple comparisons post test. The deviation value is at the level of *P* < 0.05. 

## 3. Results

### 3.1. Bioactive Marker Substances from the Herbal Formula B401

**Figure 1:**
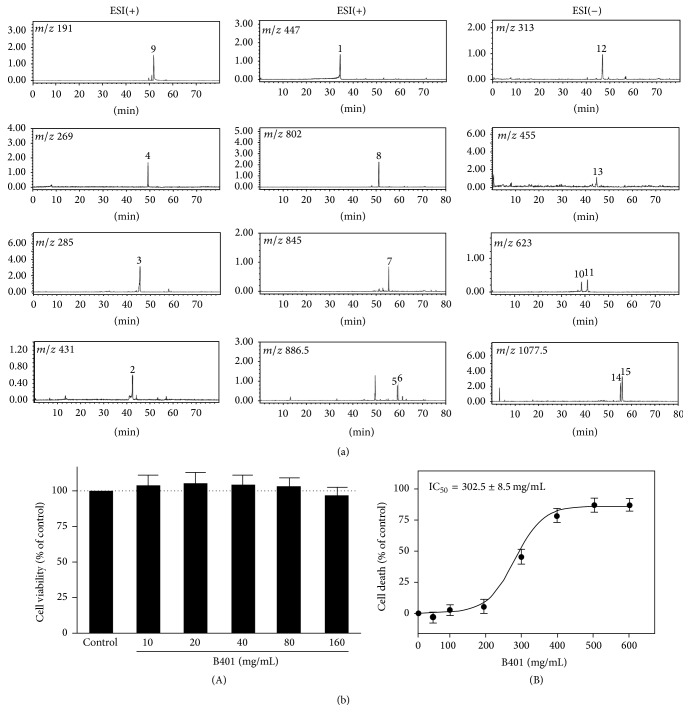
Chromatographic fingerprint analysis and cytotoxicity assay of the herbal formula B401. (a) Chromatographic fingerprint analysis was conducted by using LC/MS analysis. Fifteen bioactive marker substances from ingredients of the herbal formula B401 were qualitatively determined within 80 min under selected LC/MS condition. Bioactive marker substances for* Astragalus membranaceus*: calycosin-7-O-*β*-D-glucoside (peak 1), ononin (peak 2), calycosin (peak 3), formononetin (peak 4), astragaloside (peak 5), isoastragaloside (peak 6), astragaloside II (peak 7), and astragaloside IV (peak 8);* Angelica sinensis*: Z-ligustilide (peak 9);* Rehmannia glutinosa*: forsythiaside (peak 10) and acteoside (peak 11);* Eclipta prostrata*: wedelolactone (peak 12);* Ligustri fructus*: oleanolic acid (peak 13);* Panax ginseng*: ginsenoside Rc (peak 14) and ginsenoside Rb2 (peak 15). (b) (A) Cell viability was measured by MTT assay after Tet-On A*β*_42_-GFP SH-SY5Y cells were treated without (control) and with the herbal formula B401 at indicated doses (*n* = 6 for each treatment). (B) IC_50_ values of the herbal formula B401 for Tet-On A*β*_42_-GFP SH-SY5Y cells were reported in the dose-response curve. Results were shown as mean ± SEM, and the number of experiments was six for each treatment.

In this study, LC/MS analysis was adopted as chromatographic fingerprint analysis of the herbal formula B401 for ingredients. As shown in Figure 1(a), fifteen bioactive marker substances were qualitatively determined within 80 min under selected LC/MS condition. We observed that the herbal formula B401 mainly contains ingredients of* Astragalus membranaceus*,* Angelica sinensis*,* Rehmannia glutinosa*,* Eclipta prostrata*,* Ligustri fructus*, and* Panax ginseng*. Bioactive marker substances for* Astragalus membranaceus* were calycosin-7-O-*β*-D-glucoside in peak 1, ononin in peak 2, calycosin in peak 3, formononetin in peak 4, astragaloside in peak 5, isoastragaloside in peak 6, astragaloside II in peak 7, and astragaloside IV in peak 8; bioactive marker substances for* Angelica sinensis* were Z-ligustilide in peak 9; bioactive marker substances for* Rehmannia glutinosa* were forsythiaside in peak 10 and acteoside in peak 11; bioactive marker substances for* Eclipta prostrata* were wedelolactone in peak 12; bioactive marker substances for* Ligustri fructus* were oleanolic acid in peak 13; and bioactive marker substances for* Panax ginseng* were ginsenoside Rc in peak 14 and ginsenoside Rb2 in peak 15.

### 3.2. The IC_50_ Values of the Herbal Formula B401

As detected by MTT method, Tet-On A*β*_42_-GFP SH-SY5Y cells were treated with a series of concentrations of the herbal formula B401 from 10 to 160 mg/mL, respectively, for five days (Figure 1(b)(A)). The herbal formula B401 dose-response curve was shown in Figure 1(b)(B). Sigmoid graph shows the survival of Tet-On A*β*_42_-GFP SH-SY5Y cells at different concentrations of drug. Bars indicate the standard errors of the mean for two independently processed samples. The calculated IC_50_ values of the herbal formula B401 were 302.5 mg/mL for Tet-On A*β*_42_-GFP SH-SY5Y cells.

### 3.3. The Herbal Formula B401 Inhibits Glutamate-Induced Excitotoxicity in Tet-On A*β*_42_-GFP SH-SY5Y Cells

**Figure 2:**
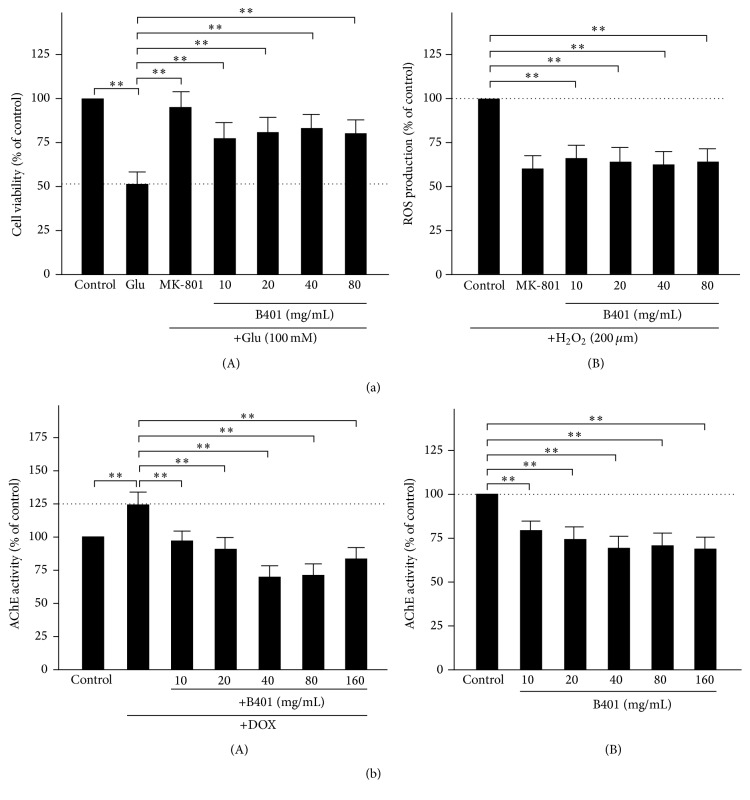
The herbal formula B401 effectively inhibits glutamate-induced excitotoxicity, H_2_O_2_-induced oxidative stress, and AChE activity in Tet-On A*β*_42_-GFP SH-SY5Y cells. (a) (A) Tet-On A*β*_42_-GFP SH-SY5Y cell viability was measured after 24 hours of glutamate-treatment (Glu, 100 mM). The herbal formulas B401 (10–80 mg/mL) and MK-801 (10 *μ*M) were added individually in the presence of glutamate. (B) Tet-On A*β*_42_-GFP SH-SY5Y cell viability was measured after 24 hours of hydrogen peroxide treatment (H_2_O_2_, 200 *μ*M). The herbal formulas B401 (10–80 mg/mL) and MK-801 (10 *μ*M) were added individually in the presence of H_2_O_2_. (b) (A) AChE activity of Tet-On A*β*_42_-GFP SH-SY5Y cells was analyzed after treating with 10 *μ*g/mL Dox in the absence or presence of the herbal formula B401 at indicated doses. (B) AChE activity of SH-SY5Y cells was analyzed in the absence or presence of the herbal formula B401 at indicated doses. Results were shown as mean ± SEM (^*∗∗*^ *P* < 0.01, two-way ANOVA followed by a Student-Newman-Keuls multiple comparisons post test), and the number of experiments was six for each treatment.

Tet-On A*β*_42_-GFP SH-SY5Y cells were treated with glutamate (100 mM) and a series of concentrations of the herbal formula B401 or MK-801 (10 *μ*M), an NMDA receptor antagonist. As shown in Figure 2(a)(A), Tet-On A*β*_42_-GFP SH-SY5Y cell viability was reduced approximately 50% compared to control by exposure to 100 mM glutamate (Glu versus control, *P* < 0.01), while it was significantly increased approximately 45% in the presence of MK-801 (Glu versus MK-801, *P* < 0.01). Results similar to MK-801 show that glutamate-treated Tet-On A*β*_42_-GFP SH-SY5Y cell viability was significantly increased approximately 30–40% in the presence of the herbal formula B401 with a series of concentrations from 10 to 80 mg/mL, respectively (Glu versus B401, *P* < 0.01).

### 3.4. The Herbal Formula B401 Inhibits H_2_O_2_-Induced Oxidative Stress in Tet-On A*β*_42_-GFP SH-SY5Y Cells

Tet-On A*β*_42_-GFP SH-SY5Y cells were treated with H_2_O_2_ (200 *μ*M) and a series of concentrations of the herbal formula B401 or MK-801 (10 *μ*M). As shown in Figure 2(a)(B), ROS production of H_2_O_2_-treated Tet-On A*β*_42_-GFP SH-SY5Y cells was reduced approximately 45% compared to control in the presence of MK-801 (control versus MK-801, *P* < 0.01). Results similar to MK-801 show that ROS production of H_2_O_2_-treated Tet-On A*β*_42_-GFP SH-SY5Y cells was significantly reduced approximately 30–35% in the presence of the herbal formula B401 with a series of concentrations from 10 to 80 mg/mL, respectively (control versus B401, *P* < 0.01).

### 3.5. The Herbal Formula B401 Inhibits AChE Activity in Tet-On A*β*_42_-GFP SH-SY5Y Cells

AChE activity of Tet-On A*β*_42_-GFP SH-SY5Y cells was expressed by A*β*_42_-GFP. Tet-On A*β*_42_-GFP SH-SY5Y cells were pretreated with various concentrations (10–160 mg/mL) of the herbal formula B401 for 24 hours and then induced with 10 *μ*g/mL Dox to express A*β*_42_-GFP for five days. As shown in Figure 2(b)(A), A*β*_42_-GFP of Tet-On A*β*_42_-GFP SH-SY5Y cells under Dox treatment was increased 25% compared to that of control group, while A*β*_42_-GFP of Tet-On A*β*_42_-GFP SH-SY5Y cells with pretreatment of the herbal formula B401 at dose of 10–160 mg/mL was significantly decreased 28–55%, respectively, comparing to that of DOX treatment. The result indicates that the herbal formula B401 effectively inhibits the AChE activity of Tet-On A*β*_42_-GFP SH-SY5Y cells at the dosage of 30-fold lower than IC_50_. We further analyzed AChE activity of SH-SY5Y cells in the absence or presence of the herbal formula B401 at indicated doses. As shown in Figure 2(b)(B), AChE activity of SH-SY5Y cells was significantly reduced approximately 25–35% in the presence of the herbal formula B401 with a series of concentrations from 10 to 160 mg/mL, respectively (control versus B401, *P* < 0.01).

### 3.6. Oral B401 Treatment Ameliorates the Deficits of Spatial Learning and Memory in 3× Tg-AD Transgenic Mice

**Figure 3:**
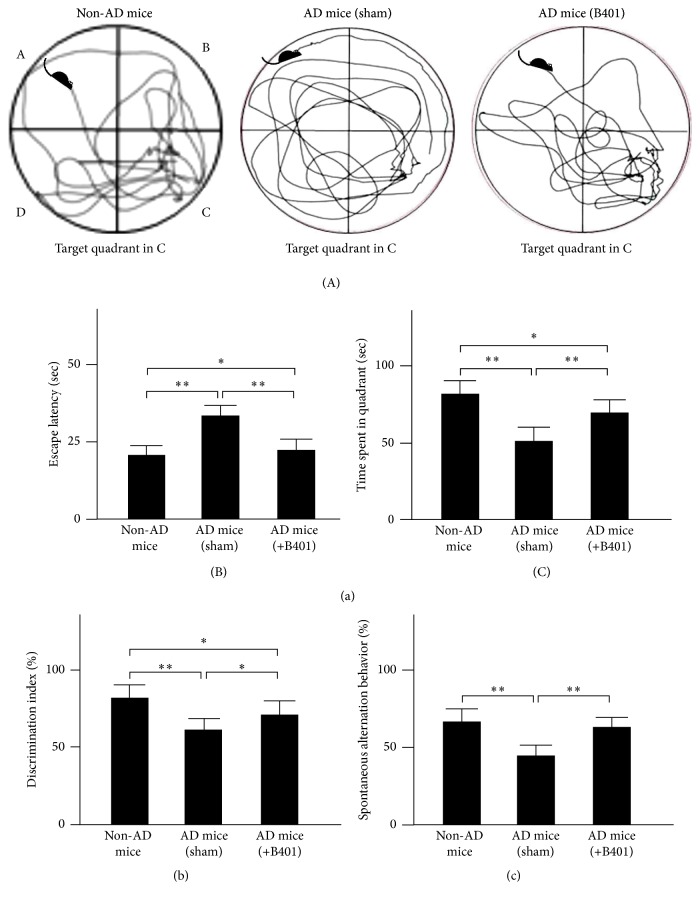
Oral treatment of herbal formula B401 effectively ameliorates cognitive dysfunction in 3× Tg-AD mice. (a) Morris water maze in 3× Tg-AD mice and their control (non-AD mice). (A) Representative path tracing experiments in 3× Tg-AD mice and their control (non-AD mice) in which mice were started from quadrant A and target in quadrant C. (B) Oral B401 treatment significantly reduces escape latency to reach the platform in 3× Tg-AD mice over 8 test days. (C) oral B401 treatment significantly increases the time spent in the target quadrant during the probe test in 3× Tg-AD mice. (b) Novel object recognition task in 3× Tg-AD mice and their control (non-AD mice). The discrimination index was calculated as percentage ratio of *T*_*B*_/(*T*_*A*_ + *T*_*B*_) × 100. *T*_*A*_: familiar object. *T*_*B*_: novel object. (c) Spontaneous alternation behavior Y-maze test in 3× Tg-AD mice and their control (non-AD mice). Alternation (%) = [(Number of alternations)/(Total arm entries − 2)] × 100. The number of mice was eight for each group. Values are mean ± SEM (^*∗∗*^ *P* < 0.01; ^*∗*^ *P* < 0.05, one-way ANOVA followed by a Student- Newman-Keuls multiple comparisons post test).

Morris water maze test was carried out to evaluate effects of the herbal formula B401 on the deficits of spatial learning and memory in 8-month 3× Tg-AD mice (Figure 3(a)(A)). We recorded the swim velocity of mice to analyze the locomotives in 3× Tg-AD mice and their control. Our results observed that 3× Tg-AD mice with sham and the herbal formula B401 treatments and their control displayed similar swim speed. Then we recorded the escape latency to reach the platform in 3× Tg-AD mice and their control (non-AD mice). As shown in Figure 3(a)(B), we found that 3× Tg-AD mice with sham treatment spent significantly longer escape latency to reach the platform than that of their non-AD mice (*P* < 0.01), while 3× Tg-AD mice with the herbal formula B401 treatment spent significantly shorter escape latency to reach the platform than that of 3× Tg-AD mice with sham treatment (*P* < 0.01). After training, the platform was removed to perform probe trial, and the path length and the time spent in quadrants were recorded in Figure 3(a)(C). Our results showed that 3× Tg-AD mice with sham treatment spent significantly shorter residence time in the target quadrant than that of their non-AD mice (*P* < 0.01), while 3× Tg-AD mice with the herbal formula B401 treatment spent significantly longer residence time than that of 3× Tg-AD mice with sham treatment (*P* < 0.01).

### 3.7. Oral B401 Treatment Ameliorates the Deficits of Short-Term Memory in 3× Tg-AD Transgenic Mice

The novel object recognition task was carried out to elucidate effects of the herbal formula B401 on the deficits of short-term memory in 8-month 3× Tg-AD mice. We recorded and compared the discrimination index of the novel object recognition task in 3× Tg-AD mice and their control in Figure 3(b). During the test phase, 3× Tg-AD mice with sham treatment did not preferentially explore the novel object, and the discrimination index is approximately 63%. It illustrated that familiar object was not completely encoded; therefore less attention was paid to the novel one. In contrast, non-AD mice and 3× Tg-AD mice with B401 treatment displayed a certain preference for the novel object, and the discrimination index is 83% and 72%, respectively.

### 3.8. Oral B401 Treatment Ameliorates the Deficits of Working Memory in 3× Tg-AD Transgenic Mice

The spontaneous alternation behavior Y-maze test was applied to observe effects of the herbal formula B401 on the deficits of working memory in 8-month 3× Tg-AD mice. We recorded and compared the percentage of alternation of the spontaneous alternation behavior Y-maze test in 3× Tg-AD mice and their control in Figure 3(b). Vehicle-treated 3× Tg-AD mice with sham treatment displayed significantly decrement of the percentage of alternation compared to that of their non-AD mice (*P* < 0.01), while vehicle-treated 3× Tg-AD mice with B401 treatment displayed significantly increase of the percentage of alternation compared to that of 3× Tg-AD mice with sham treatment (*P* < 0.01).

### 3.9. Oral B401 Treatment Alleviates Brain Atrophy and Enhances Brain BDNF Expression in 3× Tg-AD Mice

**Figure 4:**
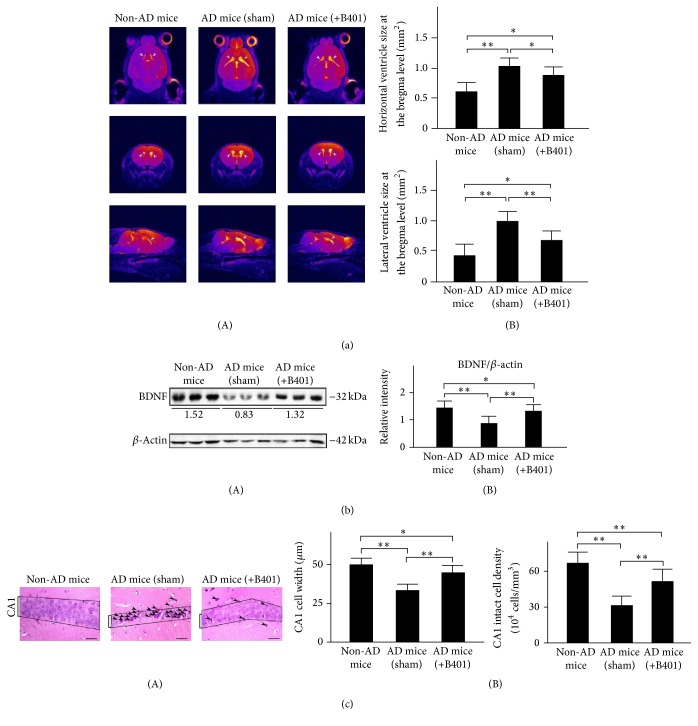
Oral B401 treatment effectively alleviates brain atrophy, enhances brain BDNF expression, and increases CA1 cell viability in 3× Tg-AD mice. (a) (A) High resolution T2w images corresponding to the brain in 3× Tg-AD mice and their control (non-AD mice). (B) Quantified horizontal and lateral ventricle sizes at the bregma level in 3× Tg-AD mice with oral B401 treatment were significantly less compared to those 3× Tg-AD mice with sham treatment but significantly greater compared to their control. (b) Western blotting analysis shows the following. (A) Whole brain BDNF expression levels of 3× Tg-AD mice and their control. (B) Quantified brain BDNF levels of the 3× Tg-AD mice with oral B401 treatment were significantly increased compared to those 3× Tg-AD mice with sham treatment but were significantly decreased compared to their control. (c) (A) H&E staining shows widths and intact cell density of hippocampal CA1 areas of 3× Tg-AD mice and their control (non-AD mice). Dead or incomplete hippocampal cells were marked with arrows. Scale bars: 30 *μ*m. (B) Quantified widths and intact cell density of hippocampal CA1 areas of the 3× Tg-AD mice with oral B401 treatment were significantly increased compared to those 3× Tg-AD mice with sham treatment but were significantly decreased compared to their control. Results were shown as mean ± SEM (^*∗∗*^ *P* < 0.01; ^*∗*^ *P* < 0.05, one-way ANOVA followed by a Student-Newman-Keuls multiple comparisons post test), and the number of experiments was six for each treatment.

Concomitant to behavioral assessments, magnetic resonance imaging (MRI) was used to noninvasively study brain structure. We observed visible brain atrophy in 8-month 3× Tg-AD mice and their control by using MRI in Figure 4(a)(A). Our MRI data reveal that 3× Tg-AD mice develop brain atrophy, while the herbal formula B401 alleviates brain atrophy in 3× Tg-AD mice. We further quantified horizontal and lateral ventricle sizes at the bregma level in the brain of 3× Tg-AD mice and their non-AD mice in Figure 4(a)(B). By computing high resolution T2w images, both quantified horizontal and lateral ventricle sizes of 3× Tg-AD mice with sham treatment were significantly greater compared to those of non-AD mice (Figure 4(a)(B), *P* < 0.01). Furthermore, both quantified horizontal and lateral ventricle sizes of 3× Tg-AD mice with B401 treatment were significantly decreased compared to those of 3× Tg-AD mice with sham treatment (Figure 4(a)(B), *P* < 0.01). Even though the herbal formula B401 significantly decreased lateral ventricle sizes in the brain of 3× Tg-AD mice, they were still significantly greater comparing to that of non-AD mice (Figure 4(a)(B), *P* < 0.05).

We used western blotting analysis to determine whether the herbal formula B401 enhances brain BDNF expression levels in 3× Tg-AD mice (Figure 4(b)(A)). The BDNF is a neurotrophin of growth factors that contributes to neuroprotection in 3× Tg-AD mice. As shown in Figure 4(b)(B), quantified brain BDNF expression levels of 3× Tg-AD mice were significantly decreased compared to those of non-AD mice (*P* < 0.01), while quantified brain BDNF expression levels of 3× Tg-AD mice with B401 treatment were significantly increased compared to those of 3× Tg-AD mice with sham treatment (*P* < 0.01). Even though the herbal formula B401 significantly increased BDNF levels in the brain of 3× Tg-AD mice, they were significantly decreased compared to that of non-AD mice (*P* < 0.05). Our results suggest that B401 treatment may effectively alleviate brain atrophy and enhance brain BDNF expression in 8-month 3× Tg-AD mice.

We further analyzed widths and intact cell density of hippocampal CA1 areas of 3× Tg-AD mice and their control (non-AD mice) from continuous brain slices with H&E staining. As shown in Figure 4(c)(B), quantified widths and intact cell density of hippocampal CA1 areas of the 3× Tg-AD mice with oral B401 treatment were significantly increased compared to those 3× Tg-AD mice with sham treatment (*P* < 0.01) but were significantly decreased compared to their control (*P* < 0.01).

### 3.10. Oral B401 Treatment Improves Subcutaneous Microcirculation and Enhances Brain VEGF Expression in 3× Tg-AD Mice

**Figure 5:**
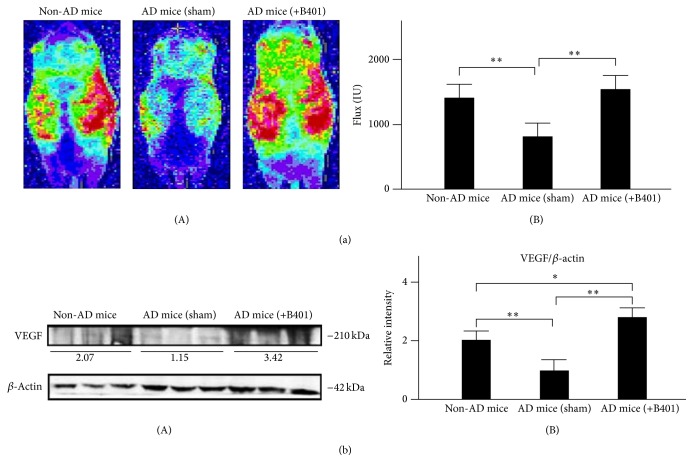
Oral B401 treatment effectively improves subcutaneous microcirculation and enhances brain VEGF expression in 3× Tg-AD mice. (a) (A) Dorsal subcutaneous microcirculatory flow imaging in 3× Tg-AD mice and their control (non-AD mice) by using moorFLPI laser Doppler imager. (B) Quantified dorsal subcutaneous microcirculatory flow in 3× Tg-AD mice with oral B401 treatment was significantly greater compared to those 3× Tg-AD mice with sham treatment. (b) Western blotting analysis shows the following. (A) Whole brain VEGF expression levels of 3× Tg-AD mice and their control. (B) Quantified brain VEGF levels of the 3× Tg-AD mice with oral B401 treatment were significantly greater compared to those 3× Tg-AD mice with sham treatment and their control. Results were shown as mean ± SEM (^*∗∗*^ *P* < 0.01; ^*∗*^ *P* < 0.05, one-way ANOVA followed by a Student-Newman-Keuls multiple comparisons post test), and the number of experiments was six for each treatment.

As suggested before, the herbal formula B401 is Taiwan-US patent TCMs in supporting healthy cardiovascular function. Thus we used the moorFLPI high resolution laser Doppler imager to noninvasively study subcutaneous microcirculatory flows in 8-month 3× Tg-AD mice and their control. As shown in Figure 5(a)(A), we observed that dorsal subcutaneous microcirculatory flow imaging of 3× Tg-AD mice with sham treatment was obviously reduced comparing to that of non-AD mice, while that of 3× Tg-AD mice with B401 treatment was obviously increased comparing to that of 3× Tg-AD mice with sham treatment. We further quantified dorsal subcutaneous microcirculatory flows in 3× Tg-AD mice and their control. As shown in Figure 5(a)(B), dorsal subcutaneous microcirculatory flows of 3× Tg-AD mice with B401 treatment were significantly greater comparing to that of 3× Tg-AD mice with sham treatment (*P* < 0.01). Similarly, dorsal subcutaneous microcirculatory flows of non-AD mice were significantly greater comparing to that of 3× Tg-AD mice with sham treatment (*P* < 0.01).

We further used western blotting analysis to determine whether the herbal formula B401 enhances brain VEGF expression levels in 3× Tg-AD mice (Figure 5(b)(A)). It has been reported that VEGF plays an important role in brain angiogenic effects [34]. As shown in Figure 5(b)(B), quantified brain VEGF expression levels of 3× Tg-AD mice with B401 treatment were significantly increased compared to that of 3× Tg-AD mice with sham treatment (*P* < 0.01). Also, quantified brain VEGF expression levels of non-AD mice were significantly increased compared to that of 3× Tg-AD mice with sham treatment (*P* < 0.01). Our results suggest that B401 treatment may effectively improve subcutaneous blood flow and enhance brain VEGF expression in 8-month 3× Tg-AD mice.

### 3.11. Oral B401 Treatment Reduces Blood ROS and Inhibits Brain Oxidative Stress in 3× Tg-AD Mice

**Figure 6:**
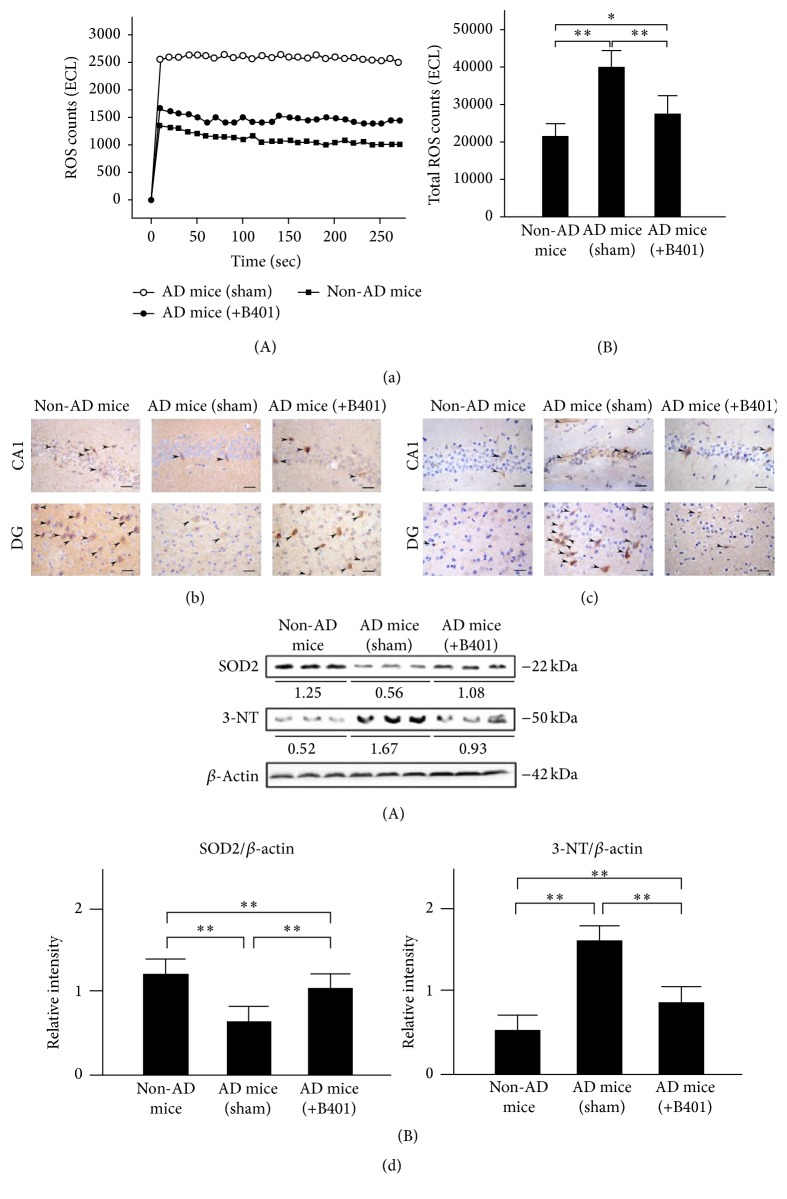
Oral B401 treatment effectively reduces blood ROS and enhances SOD2 but decreases 3-NT expressions in the brain of 3× Tg-AD mice. (a) (A) Blood ROS corresponding to the brain in 3× Tg-AD mice and their control (non-AD mice) by using chemiluminescence analysis. (B) Quantified blood ROS in 3× Tg-AD mice with oral B401 treatment were significantly decreased compared to those 3× Tg-AD mice with sham treatment but were greater compared to their control. (b) IHC staining shows SOD2 expression (marked by arrows) and (c) 3-NT expression (marked by arrows) in CA1 and dentate gyrus areas of 3× Tg-AD mice and their control (non-AD mice). Scale bars: 30 *μ*m. (d) Western blotting analysis shows the following. (A) Whole brain SOD2 and 3-NT expression levels of 3× Tg-AD mice and their control. (B) Quantified brain SOD2 levels of the 3× Tg-AD mice with oral B401 treatment were significantly increased, but brain 3-NT levels were significantly decreased compared to those 3× Tg-AD mice with sham treatment. Results were shown as mean ± SEM (^*∗*^ *P* < 0.05; ^*∗∗*^ *P* < 0.01, one-way ANOVA followed by a Student-Newman-Keuls multiple comparisons post test), and the number of experiments was six for each treatment.

To explored whether oral B401 treatment affects oxidative stress in 3× Tg-AD mice and their control, we examined blood ROS levels by using a CLA-ID3 chemiluminescence analyzer. As shown in Figure 6(a), blood ROS levels of 3× Tg-AD mice with sham treatment were greatly increased when compared with those of non-AD mice and 3× Tg-AD mice with B401 treatment. Total counts of blood ROS were further quantified in Figure 6(a)(B). We found that total counts of blood ROS of 3× Tg-AD mice with B401 treatment were significantly reduced compared to that of 3× Tg-AD mice with sham treatment (*P* < 0.01), even though total counts of blood ROS of 3× Tg-AD mice with B401 treatment were still significantly increased compared to that of non-AD mice (*P* < 0.05).

SOD2 is a marker of antioxidant enzyme, while 3-NT is a marker of oxidative damage [35]. Thus we also compared SOD2 and 3-NT in the brains of 3× Tg-AD mice and their control by IHC staining and western blotting analysis. As observed from IHC staining of the brain, in comparison to non-AD mice, SOD2 expressions were not obvious (Figure 6(b)), but 3-NT expressions were obvious (Figure 6(c)) in 3× Tg-AD mice with sham treatment. In comparison to 3× Tg-AD mice with sham treatment, SOD2 expressions were obvious (Figure 6(b)), while 3-NT expressions were not obvious (Figure 6(c)) in hippocampal CA1 and dentate gyrus (DG) areas of 3× Tg-AD mice with B401 treatment. Quantified SOD2 levels in 3× Tg-AD mice with sham treatment were significantly reduced compared to those of 3× Tg-AD mice with B401 treatment and their control of non-AD mice (Figure 6(d)(B), *P* < 0.01), even though quantified SOD2 levels of 3× Tg-AD mice with B401 treatment were significantly reduced compared to those of non-AD mice (Figure 6(d)(B), *P* < 0.01). On the contrary, quantified 3-NT levels in 3× Tg-AD mice with sham treatment were significantly increased compared to those of 3× Tg-AD mice with B401 treatment and their control of non-AD mice (Figure 6(d)(B), *P* < 0.01), even though quantified 3-NT levels of 3× Tg-AD mice with B401 treatment were significantly increased when compared with their control of non-AD mice (Figure 6(d)(B), *P* < 0.01).

### 3.12. Oral B401 Treatment Inhibits Brain Expressions of A*β*, Phosphorylated Tau, and NFTs in 3× Tg-AD Mice

**Figure 7:**
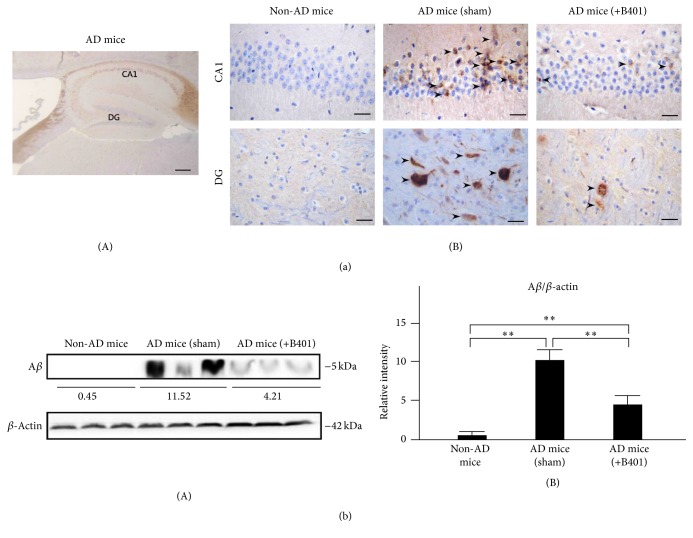
Oral B401 treatment effectively inhibits brain A*β* expression in 3× Tg-AD mice. (a) IHC staining shows A*β* expressions (marked by arrows) in hippocampal CA1 and dentate gyrus (DG) areas of 3× Tg-AD mice and their control (non-AD mice). Scale bars: 30 *μ*m. (b) Western blotting analysis shows the following. (A) Whole brain A*β* expression levels of 3× Tg-AD mice and their control; (B) Quantified brain A*β* levels of 3× Tg-AD mice with oral B401 treatment were significantly less than those 3× Tg-AD mice with sham treatment but were significantly greater than their control. Results were shown as mean ± SEM (^*∗∗*^ *P* < 0.01, one-way ANOVA followed by a Student-Newman-Keuls multiple comparisons post test), and the number of experiments was six for each treatment.

**Figure 8:**
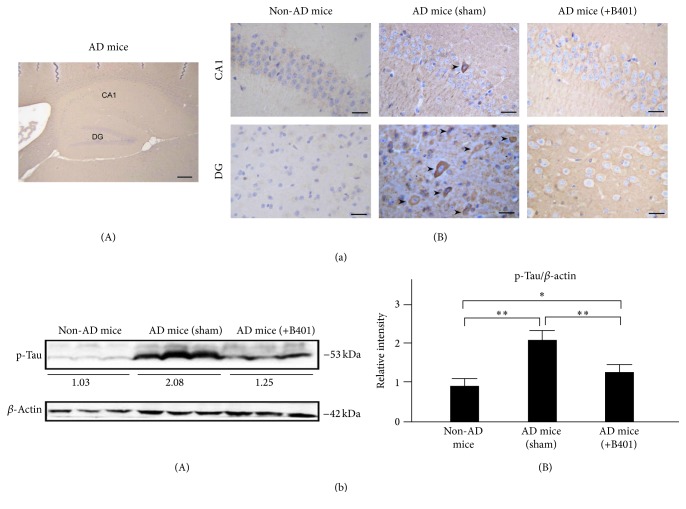
Oral B401 treatment effectively inhibits brain p-Tau expression in 3× Tg-AD mice. (a) IHC staining shows p-Tau expression (marked by arrows) in hippocampal CA1 and dentate gyrus (DG) areas of 3× Tg-AD mice and their control (non-AD mice). Scale bars: 30 *μ*m. (b) Western blotting analysis shows the following. (A) Whole brain p-Tau expression levels of 3× Tg-AD mice and their control. (B) Quantified brain p-Tau levels of 3× Tg-AD mice with oral B401 treatment were significantly less than those 3× Tg-AD mice with sham treatment but were significantly greater than their control. Results were shown as mean ± SEM (^*∗∗*^ *P* < 0.01; ^*∗*^ *P* < 0.05, one-way ANOVA followed by a Student-Newman-Keuls multiple comparisons post test), and the number of experiments was six for each treatment. AD: Alzheimer's disease; CA1: region 1 of hippocampus proper; DG: dentate gyrus; IHC: immunohistochemistry; SEM: standard error of the mean.

**Figure 9:**
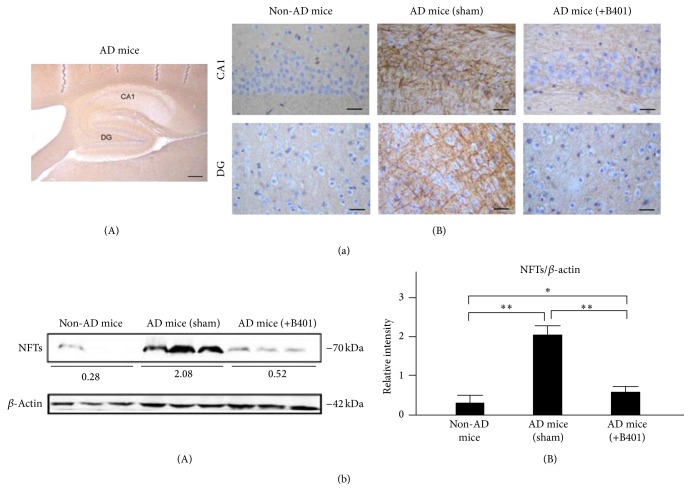
Oral B401 treatment effectively inhibits brain NFTs expression in 3× Tg-AD mice. (a) IHC staining shows NFTs expression (marked by brown color) in hippocampal CA1 and dentate gyrus (DG) areas of 3× Tg-AD mice with and their control (non-AD mice). Scale bars: 30 *μ*m. (b) Western blotting analysis shows the following. (A) Whole brain NFTs expression levels of 3× Tg-AD mice and their control. (B) quantified brain NFTs levels of 3× Tg-AD mice with oral B401 treatment were significantly less than those 3× Tg-AD mice with sham treatment but were significantly greater than their control. Results were shown as mean ± SEM (^*∗∗*^ *P* < 0.01; ^*∗*^ *P* < 0.05, one-way ANOVA followed by a Student-Newman-Keuls multiple comparisons post test), and the number of experiments was six for each treatment.

As a result of immunohistochemical (IHC) analysis shown in Figures 7(a), 8(a), and 9(a), we examined A*β*, phosphorylated Tau, and NFTs expressions in hippocampus CA1 and DG areas. Our results found that IHC staining expressions of A*β*, phosphorylated Tau, and NFTs were quite not obvious in CA1 and DG areas of non-AD mice. On the contrary, IHC staining expressions of A*β*, phosphorylated Tau, and NFTs in CA1 and DG areas of 3× Tg-AD mice with sham treatment were quite obvious. Unlike results of 3× Tg-AD mice with sham treatment, we observed that IHC staining expressions of A*β*, phosphorylated Tau, and NFTs became indistinct in CA1 and DG areas of 3× Tg-AD mice with B401 treatment (Figures 7(a)(B), 8(a)(B), and 9(a)(B)).

As analyzed from western blotting analysis, quantified brain A*β*, phosphorylated Tau, and NFTs expression levels of 3× Tg-AD mice were significantly greater than those of non-AD mice (Figures 7(b)(B), 8(b)(B), and 9(b)(B), *P* < 0.01), while quantified brain A*β*, phosphorylated Tau, and NFTs expression levels of B401-treated 3× Tg-AD mice were significantly decreased compared to those of 3× Tg-AD mice with sham treatment (Figures 7(b)(B), 8(b)(B), and 9(b)(B), *P* < 0.01). Even though the herbal formula B401 significantly decreased A*β*, phosphorylated Tau, and NFTs levels in the brain of 3× Tg-AD mice, they were significantly increased comparing to those of non-AD mice (Figures 7(b)(B), 8(b)(B), and 9(b)(B), *P* < 0.01–0.05).

## 4. Discussion

The present study demonstrates that the herbal formula B401 has neuroprotective effects on Tet-On A*β*_42_-GFP SH-SY5Y cells because the herbal formula B401 treatment inhibits glutamate-induced excitotoxicity and AChE activity. Furthermore, the present study clarifies that the herbal formula B401 has neuroprotective effects on 3× Tg-AD mice because oral herbal formula B401 treatment effectively ameliorates neurocognitive dysfunction in 3× Tg-AD mice via Morris water maze test, spontaneous alternation behavior Y-maze test, and novel object recognition task. In addition, oral herbal formula B401 treatment effectively alleviates brain atrophy and enhances brain BDNF expression, improves subcutaneous blood flow and enhances brain VEGF expression, and reduces blood ROS and enhances brain SOD2 expression but reduces brain 3-NT expression in 3× Tg-AD mice. Particularly, the present study demonstrates that oral herbal formula B401 treatment effectively reduces expressions of amyloid beta, phosphorylated Tau, and neurofibrillary tangles in the brain of 3× Tg-AD mice.

As reported in previous studies, there is much attention towards herbal remedy of many brain disorders such as AD, Parkinson's disease, and Huntington's disease [36–38]. Here we found the herbal formula B401 has alternative medical applications in both AD cell and mouse models. By MTT assay, quite less cytotoxicity of the herbal formula B401 was detected in Tet-On A*β*_42_-GFP SH-SY5Y cells (Figure 1(b)). The herbal formula B401 was found to inhibit glutamate-induced excitotoxicity and H_2_O_2_-induced oxidative stress in Tet-On A*β*_42_-GFP SH-SY5Y cells (Figure 2(a)). As suggested in previous studies, many bioactive substances from the herbal formula B401 have been verified with neuroprotective properties. For example, ginsenoside Rb1 and formononetin have been used to protect brain function by suppressing cellular excitotoxicity [39, 40]. The herbal formula B401 only inhibits glutamate-induced excitotoxicity in previous study, but it was also found to inhibit AChE activity in SH-SY5Y cells and Tet-On A*β*_42_-GFP SH-SY5Y cells induced by Dox to express A*β*_42_-GFP in this study (Figure 2(b)). Nowadays, there is no effective treatment for AD, although AChE inhibitors can attenuate AD symptoms, especially on dementia; they have limitation on improvement of the progression of the disease and side effects [19].

3× Tg-AD mice overexpress that human APPswe, human tauP301L, and human PS1M146V mutations are generated to mimic the pathology of AD. According to previous studies, 3× Tg-AD mice exhibit progressive cognitive impairments as early as 4-5 months prior to A*β* deposition, and the performance on the Morris water maze displays statistic difference after 6 months [26–28]. In this study, we observed that 6-month 3× Tg-AD mice have exhibited significantly deficits of learning and memory, while chronic treatment with the herbal formula B401 for 2 months significantly attenuated spatial learning and memory of 8-month 3× Tg-AD mice via Morris water maze test (Figure 3(a)). Also, 2-month oral treatment with the herbal formula B401 effectively ameliorates the deficits of short-term memory in 8-month 3× Tg-AD mice via the novel object recognition task (Figure 3(b)). Furthermore, we also found that 2-month oral B401 treatment effectively ameliorates the deficits of working memory in 8-month 3× Tg-AD mice via the spontaneous alternation behavior Y-maze test (Figure 3(c)).

In the early stage of AD, the synapse loss in the neocortex and hippocampus is robustly correlated with cognitive deficits and dementia [14, 41, 42]. Addition of A*β*1–42 to primary cortical and hippocampal neurons obstructs synapse formation and neurite outgrowth [43]. Here we observed visible brain atrophy in 8-month 3× Tg-AD mice by using high resolution T2w images (Figure 4(a)(A)). As suggested before, the herbal formula B401 is widely used as a health supplement in supporting healthy brain and cardiovascular function. The present study demonstrated that oral treatment with the herbal formula B401 had significant alleviation on brain atrophy in 8-month 3× Tg-AD mice (Figure 4(a)(B)). Similarly, our H&E staining results demonstrated that oral treatment with the herbal formula B401 significantly increased widths and intact cell density of hippocampal CA1 areas of the 3× Tg-AD mice (Figure 4(c)).

Moreover, oral treatment with the herbal formula B401 had significant enhancement on subcutaneous microcirculation in 8-month 3× Tg-AD mice (Figure 5(a)). As reported in previous study, AD individuals have been found decreased BDNF expressions in the hippocampus, while enhanced BDNF expressions may have neuroprotective effects on the hippocampus of AD individuals [25]. Furthermore, it was also reported that VEGF may prevent A*β*-induced endothelial apoptosis* in vitro*. Neuronal expression of VEGF in transgenic AD mice could restore memory behavior [44]. To support these previous studies, the present study demonstrated that oral treatment with the herbal formula B401 may alleviate brain atrophy of 8-month 3× Tg-AD mice via enhancing brain BDNF expressions (Figure 4(b)) and improve subcutaneous microcirculation of 8-month 3× Tg-AD mice via enhancing brain VEGF expressions (Figure 5(b)).

Oxidative stress plays a crucial role in the pathogenesis of AD that activated microglia, A*β* plaques, and NFTs [45–47]. In the present study, we found that oral herbal formula B401 treatment may suppress the brain oxidative stress via suppressing ROS generation in 8-month 3× Tg-AD mice (Figure 6(a)). As described before, SOD2 is an important antioxidant enzyme for oxidative stress, while 3-NT is a marker of oxidative damage [35]. We further found that oral herbal formula B401 treatment may enhance SOD2 and reduce 3-NT expressions in the brain of 3× Tg-AD mice, especially in hippocampal CA1 and DG areas (Figures 6(b) and 6(c)). Our results may provide evidence that the herbal formula B401 could protect AD pathogenesis through antioxidant property in the brain of 3× Tg-AD mice.

As described in previous study, 3× Tg-AD mice are generated to mimic the pathology of AD and successfully developed both amyloid plaque and NFTs-like pathology [26]. Extracellular amyloid plaques are detectable at 6 months in the frontal cortex and hippocampus [27]. Furthermore, A*β* accumulation correlates to the increase of Tau protein phosphorylation and NFTs. In the present study, we observed amyloid burden, phosphorylated Tau, and NFTs in the cortex and hippocampus of 8-month-old 3× Tg-AD mice (Figures 7(a), 8(a), and 9(a)). On the contrary, we found that oral herbal formula B401 treatment may reduce A*β*, phosphorylated Tau, and NFTs expressions in the brain of 3× Tg-AD mice, especially in hippocampal CA1 and DG areas (Figures 7, 8, and 9). The results may provide evidence that the herbal formula B401 could protect AD pathogenesis through antiamyloidogenesis, and anti-Tau phosphorylation, and aggregation in the brain of 3× Tg-AD mice.

## 5. Conclusions

**Figure 10:**
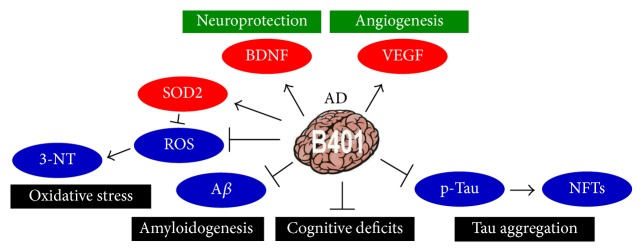
The schematic diagram illustrates the possible neuroprotective pathways of B401 treatment in the brain tissue of 3× Tg-AD mice. 3× Tg-AD mice under oral herbal formula B401 treatment enhance neuroprotection via increasing expression levels of BDNF, VEGF, and antioxidative SOD2 and alleviate neurodegeneration via suppressing ROS production, decreasing expression levels of A*β*, p-Tau, NFTs, and oxidation-related 3-NT. Taking these effects, the herbal formula B401 may alleviate cognitive deficits in 3× Tg-AD mice.

Here we used the herbal formula B401, a Taiwan-US patent Chinese herbal medicine, as alternative medical applications in remission of AD-induced neurotoxicity. As summarized in Figure 10, the present study reported that oral B401 treatment significantly improves cognitive abilities such as spatial learning and memory as well as short-term memory in 3× Tg-AD mice. In addition, oral B401 treatment may have neuroprotective effects on the brain of 3× Tg-AD mice via increasing expressions of BDNF, VEGF, and antioxidative SOD2, while suppressing ROS production and reducing expressions of A*β*, p-Tau, NFTs, and oxidation-related 3-NT. It has been clarified that AD was induced via multiple pathological or neurotoxic pathways. As suggested from our results and previous study, we found that the herbal formula B401 has multifunction in blood circulation activation and neurodegenerative protection in AD and HD transgenic mice [20, 21]. Thus we suggested that the herbal formula B401 may have the potential to be developed into an optimum TCM for neurodegenerative diseases such as AD and HD.

## Abbreviations

AChE: Acetylcholinesterase
AD: Alzheimer's disease
AU: Arbitrary perfusion units
A*β*: Amyloid beta
BDNF: Brain-derived neurotrophic factor
CA1: Region 1 of hippocampus proper
DG: Dentate gyrus
ECL: Electrochemiluminescence
ESI: Electrospray ionisation
*m/z*: Mass to charge ratio
IHC: Immunohistochemistry
LC/MS: Liquid chromatography-mass spectrometry
NFTs: Neurofibrillary tangles
NMDA: N-Methyl-D-aspartate
ROS: Reactive oxygen species
SOD2: Superoxide dismutase 2
VEGF: Vascular endothelial growth factor
3-NT: 3-Nitrotyrosine.
